# Inhibitory Effect of High Temperature- and High Pressure-Treated Red Ginseng on Exercise-Induced Oxidative Stress in ICR Mouse

**DOI:** 10.3390/nu6031003

**Published:** 2014-03-07

**Authors:** Seok-Yeong Yu, Bo-Ra Yoon, Young-Jun Lee, Jong Seok Lee, Hee-Do Hong, Young-Chul Lee, Young-Chan Kim, Chang-Won Cho, Kyung-Tack Kim, Ok-Hwan Lee

**Affiliations:** 1 Department of Food Science and Biotechnology, Kangwon National University, Chuncheon 200-701, Korea; E-Mails: dbtjrdud@naver.com (S.-Y.Y.); yoonboll@naver.com (B.-R.Y.); hslyj02@gmail.com (Y.-J.L.); jongseoklee78@gmail.com (J.S.L.); 2 Korea Food Research Institute, Gyeonggi 463-746, Korea; E-Mails: honghd@kfri.re.kr (H.-D.H.); yclee@kfri.re.kr (Y.-C.L.); yckim@kfri.re.kr (Y.-C.K.); cwcho@kfri.re.kr (C.-W.C.); tack@kfri.re.kr (K.-T.K.)

**Keywords:** red ginseng, enzyme activity, oxidative stress, glycogen contents, MDA levels

## Abstract

As previously reported, high temperature- and high pressure-treated red ginseng (HRG) contain higher contents of phenolic compounds and protect C2C12 muscle cells and 3T3-L1 adipocytes against oxidative stress. This study investigated the effect of HRG on oxidative stress using a mouse model. Our results show that the levels of glutamic oxaloacetic transaminase and glutamic pyruvic transaminase, hepatic malondialdehyde in the HRG group were significantly lower than those of the exercise groups supplemented with commercial red ginseng (CRG) or not supplemented. The muscular glycogen level, glucose-6-phosphate dehydrogenase and lactate dehydrogenase activities of the HGR group were higher than that of the CGR group. Furthermore, the HRG treatment group displayed upregulated mRNA expression of Cu/Zn-SOD and muscle regulatory factor 4. These results indicate that HRG may protect oxidative stress induced by exercise as well as improve exercise performance capacity.

## 1. Introduction

In general, appropriate exercise is helpful to prevent cancer, diabetes, stroke, and hypertension, but the habit of excessive training promotes the generation of reactive oxygen species (ROS), and then leads to oxidative stress [1]. ROS, by-products of the normal metabolism of mitochondria, are reactive molecules with at least one unpaired electron. ROS play a key role in homeostasis, signal transduction and cell proliferation in biological systems, but the amount of ROS is gradually increased through chain reactions, and the augmented concentration of the product is highly toxic in the body [2]. In addition, these products are related to lipid peroxide, protein modification, necrosis and apoptosis [3]. In order to protect the body from oxidative stress, there are endogenous enzymatic antioxidants such as superoxide dismutases (SODs), catalase and glutathione peroxidase (GPx), which attenuate the deleterious effects of ROS [4]. These antioxidants are located in intracellularly, extracellularly and in the mitochondria, and inhibit cell damage induced by ROS and free radicals in the body [5].

Ginseng (*Panax ginseng* C.A. Meyer, Araliaceae Family) is a popular traditional herbal medicine that has been used for centuries in Korea, China, and Japan. Over 30 different ginsenosides from saponin in ginseng have been isolated and identified. Major ginsenosides can be separated into protopanaxadiol-type (Rb1, Rb2, Rb3, Rc, Rd and Rg3) and protopanaxatriol-type (Re, Rg1, Rg2 and Rh1). Korean red ginseng (KRG) is processed through steaming and drying processes, and has some distinct ginsenosides, such as Rg3, Rg5, Rh1 and Rk1 [6,7,8]. Previous studies have demonstrated that distinct compounds in KRG have potent biological activities such as anti-diabetic, anti-allergic and anti-obesity activities [9,10,11]. In addition, these unique compounds of KRG are produced by heat processing, and the constituents in KRG can vary in accordance with how the conditions are applied to make KRG [12]. An important aspect is that the difference between constituents in KRG can result in different biological activities of KRG. As reported in a previous study, the high temperature and high pressure-treated red ginseng (HRG) was produced at high temperature (140 °C) and high pressure (3 kg/cm^2^) [13]. The HRG contained higher phenolic acids content, such as maltol (6213.2 μg/100 g), *p*-hydroxybenzoic acid (823.3 μg/100 g) and syringic acid (6938.8 μg/100 g) than that found in white ginseng or commercial red ginseng (CRG) [13]. Furthermore, the change of phenolic acids in HRG leads to the inhibitory effect on oxidative stress in C2C12 muscle cells and 3T3-L1 adipocytes by upregulation of the mRNA expression levels of copper and zinc (Cu/Zn)-SOD and catalase [14].

Therefore, to elucidate the inhibitory effect of HRG on oxidative stress, we investigated the oxidative stress indices in mice treated with HRG.

## 2. Materials and Methods

### 2.1. Materials

Anthrone, potassium chloride (KCl), 1,1,3,3-tetraethoxypropane (TEP), potassium hydroxide, potassium phosphate, sodium chloride (NaCl), β-nicotinamide adenine dinucleotide phosphate hydrate (β-NADP), β-nicotinamide adenine dinucleotide reduced disodium salt hydrate (NADH), glucose-6-phosphate, bradford reagent, maleimide, glucose, and glacial acetic acid were purchased from Sigma (St. Louis, MO, USA). Ethylenediaminetetraacetic acid (EDTA), bovine serum albumin, and trizma base were obtained from Bio-Rad (Bio-Rad, Hercules, CA, USA). Pyruvate was purchased from Fluka AG (Buchs, Switzerland).

### 2.2. Preparation of High Temperature- and High Pressure-Treated Red Ginseng Extract

The red ginseng treated with high temperature and high pressure was produced according to the method developed by Korea Food Research Institute. Four-year-old ginsengs were first dried at 50 °C, and then again steamed at high temperature (140 °C) and high pressure (3 kg/cm^2^) for 20 min. The steamed HRG was dried at 50 °C, and the dried HRG was ground to 20~30 mesh, and refluxed with 10 volumes (v/w) of hot water at 80 °C for 3 h. After reflux, the extract was filtered two times and concentrated at 55 °C. Then, the concentrate was freeze-dried and stored at −20 °C until used.

HPLC was used to determine phenolic acids in HRG, which contain maltol (6213.2 μg/100 g), *p*-hydroxybenzoic acid (823.3 μg/100 g), caffeic acid + vanillic acid (307.8 μg/100 g), syringic acid (6938.8 μg/100 g), *p*-coumaric acid (901.2 μg/100 g), ferulic acid (3796.7 μg/100 g), and cinnamic acid (3737.1 μg/100 g). Phenolic compounds were substantially increased compared with CRG and white ginseng as described previously [13].

### 2.3. Experimental Animals

All experimental procedures were approved by Kangwon National University Institutional Animal Care and Use Committee (IRB number; KW-12091201). Male ICR mice were purchased at three weeks-old from DooYeol Biotech (Seoul, Korea). The animals were housed in individual cages and maintained at 23–25 °C with 50%–60% relative humidity. Feed and water were provided *ad libitum* and weighed periodically during the experimental period (Table 1 and Table 2).

At the age of four weeks, the mice were randomly divided into four groups; no exercise group (CON), high intensity exercise group (E), high intensity exercise group supplemented with commercial red ginseng extract (E + CRG), high intensity exercise group supplemented with high temperature and high pressure-treated red ginseng extract (E + HRG); each group contained eight mice.

High intensity exercise was induced by a treadmill for three weeks. As shown in Table 3, the mice in the high intensity exercise groups were familiarized with the treadmill running on a motorized treadmill by the first week. The running speed and durations were determined at 5 m/min, 5 min for the first day, with an increment of 5 m/min and 5 min/day until reaching 20 m/min for 20 min without administration of HRG or GRG. After the familiarization, the mice in the high intensity exercise groups were put on the treadmill with running at 20 m/min, 20 min with or without administration of CRG (100 mg/kg BW) or HRG (100 mg/kg BW) for two weeks. Blood, liver, spleen, kidney, testicle, gastrocnemius and testicle fat were collected immediately after the last exercise. The blood was centrifuged at 7000× *g* for 10 min to separate the serum, and then stored at −70 °C for analysis. The tissues were washed with 0.9% NaCl, and then stored at −70 °C until used.

**Table 1:** Experimental design.

(*n* = 8)	Exercise	Type of Diet
CON ^1^	X	AIN-93G diet
E ^2^	O	AIN-93G diet
E + CRG ^3^	O	AIN-93G diet + CRG (100 mg/kg BW)
E + HRG ^4^	O	AIN-93G diet + HRG (100 mg/kg BW)

^1^ CON: Control; ^2^ E: Exercise; ^3^ CRG: Commercial red ginseng; ^4^ HRG: High-temperature and high pressure-treated red ginseng.

**Table 2:** Composition of experimental diets (in g/kg diet).

Formula	g/kg
Casein	200
Sucrose	100
Corn starch	397.486
l-cystein	3
Cellulose	50
Mineral mix, AIN-93G-MX (94046)	35
Vitamin mix AIN-93-VX (94047)	10
Choline Bitartrate	2.5
TBHQ ^1^, antioxidant	0.014
94045 AIN-93G Purified Diet (Harlan)	

^1^ TBHQ: *t*-Butylhydroquinone.

**Table 3:** Treadmill exercise for adaptation.

Day	1	2	3	4	5
speed (m/min)	5	10	15	20	20
time (min)	5	10	15	20	20
slope (°)	7	7	7	7	7

### 2.4. Tissue Extraction for Enzyme Activity

The gastrocnemius tissues were placed in vials, and 2 mL of 50 mM potassium phosphate buffer (pH 7.5) containing 0.15 M NaCl and 1 mM EDTA were added to the vials. The tissues were homogenized at 2000× *g* for 5 min using a tissue grinder (SolGent Co., Ltd., Daejeon, Korea). The ground tissues were centrifuged at 12,000× *g* for 15 min at 4 °C. The each supernatant was placed in new vials and stored at −20 °C until used.

### 2.5. Total Protein Assay

The protein content was based on the method of Bradford [15]. Three milliliter of Bradford reagent was added to 100 μL tissue extract. After vortexing, the absorbance of the reactants was monitored at 595 nm within 60 min. The calibration curve (*y* = 0.3633*x* + 0.004) was prepared using BSA.

### 2.6. Blood Analysis

The activities of glutamic oxaloacetic transaminase (GOT) and glutamic pyruvic transaminase (GPT) in blood serum were analyzed by AST and ALT kit (Roche, Mannheim, Germany). One milliliter of each substrate solution of GOT and GPT was placed in a tube, incubated at 37 °C for 5 min, and then 200 μL of serum was added. The GOT mixture was incubated for 60 min and the GPT mixture was incubated for 30 min, respectively. Then, color reagent was added to the mixture, vortexed and incubated at room temperature for 20 min. After incubation, 10 mL of 0.4 N NaOH was added, vortexed, and incubated for 10 min, and then the absorbance of mixture against distilled water was monitored at 505 nm. GOT and GPT activities in serum were expressed as U/L, respectively.

### 2.7. Determination of Hepatic Malondialdehyde (MDA)

Hepatic MDA content was measured by the method of Ohkawa *et al.* [16] with some modification. 100 mg of liver was accurately weighed followed the addition of 900 μL KCl (1.15%, w/v), and then sufficiently homogenized using a tissue grinder (SolGent Co., Ltd., Daejeon, Korea). The homogenate (100 μL) was placed in new tube, and 0.2 mL of 8.1% sodium dodecyl sulfate and 3.0 mL of 0.4% thiobarbituric acid (w/v) in glacial acetic acid (pH 4, 20%, v/v) were added and heated at 97 °C for 30 min. After cooling in ice, 1.0 mL of distilled water and the mixture of n-butanol:pyridine (15:1, v/v) were added to the tube and centrifuged at 3000 rpm for 15 min. The absorbance of the supernatant was monitored at 550 nm. The calibration curve (*y* = 3.8167*x* + 0.0014) was prepared using 1,1,3,3-tetraethoxypropane (TEP) and expressed as nmol MDA/g.

### 2.8. Determination of Muscular Glycogen

Muscular glycogen content was determined by the method of Seifer *et al.* [17]. 100 mg of the muscle was placed in a tube, 2 mL of KOH (30%, w/v) was added and homogenized using a tissue grinder (SolGent Co., Ltd., Daejeon, Korea). Then, the tube was heated at 100 °C for 20 min in water bath. After 20 min, 2.5 mL of ethanol was added to the tube and again heated at 100 °C for 5 min. The tube was cooled in ice, centrifuged at 3000 rpm for 15 min, the supernatant removed, and added to 1 mL of distilled water. The solution in the tube was used for measurement of muscular glycogen. One milliliter of the solution was placed in another tube, and 4 mL of anthrone reagent (0.2%, w/v) in concentrated H_2_SO_4_ was added. The tube was heated at 100 °C for 10 min, and then the absorbance of the mixture was monitored at 620 nm. The calibration curve (*y* = 1.6136*x* − 0.0211) was prepared using glucose and expressed as mg/g.

### 2.9. Measurement of Enzyme Activity in Gastrocnemius Tissue

G6PDH activity of the tissue was determined as described by Deutsch [18] with some modification. The assay medium contained 5.88 μM β-NADP, 88.5 μM MgCl_2_, 53.7 μM glucose-6-phosphate and 0.77 mM maleimide. The 50 μL of tissue extracts was added to 1 mL of the assay medium to initiate the reaction. The assay medium was used to obtain baseline of the spectrophotometer at 340 nm.

LDH activity of the tissue was measured by the method of Narang [19]. The assay mixture contained 0.1 M phosphate buffer (pH 7.5), 80 μM NADH and 0.6 mM sodium pyruvate. The assay medium was used to obtain baseline of the spectrophotometer at 340 nm and added to 20 μL of tissue extracts to initiate the oxidation. Enzyme activity was expressed in terms of μM/min per mg.

### 2.10. RNA Isolation and Semi-Quantitative RT-PCR

Total RNA was isolated from gastrocnemius tissues as described previously [14]. First, RNA purity was determined by an OD260/OD280 ratio, and one microgram of total RNA used to produce cDNA using a RT-PCR system. The density of the PCR products was normalized against the density of GAPDH from the same cDNA sample. The sequence of the oligonucleotide primer is shown in Table 4.

**Table 4:** Oligonucleotide primers used for RT-PCR.

Item	Direction	Sequence(5′-3′)
GAPDH ^1^	F ^2^	5′-CAAGGTCATCCATGACAACT-3′
	R ^3^	5′-GGCCATCCACAGTCTTCTGG-3′
Cu/Zn SOD	F	5′-CAGCATGGGTTCCACGTCCA-3′
	R	5′-CACATTGGCCACACCGTCCT-3′
MRF4	F	5′-CTGACTGGGCAGTCGGGTG-3′
	R	5′-ATGGACCTTTTTGAAACTGG-3′

^1^ GAPDH: Glyceraldehyde 3-phosphate dehydrogenase; ^2^ F: Forward; ^3^ R: Reverse.

### 2.11. Statistical Analysis

Data were expressed as means ± standard error (SEM), and the results were taken from at least three independent experiments performed in triplicate. The data were analyzed by one-way analysis of variance (ANOVA) procedure of Statistical Analysis System [20] with Duncan’s multiple-range tests for individual group comparisons. Values with different superscript letters are significantly different at the *p* < 0.05 level.

## 3. Results

### 3.1. Weight Gain and Feed Efficiency

**Figure 1:**
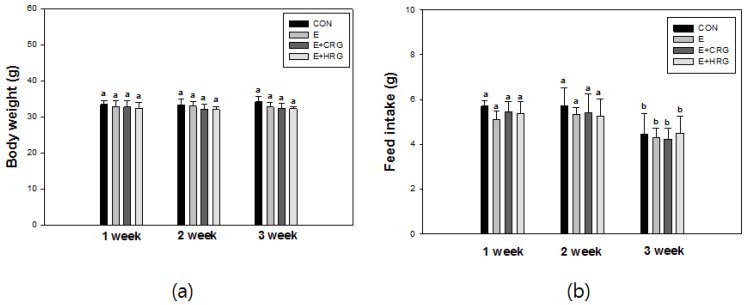
Changes of body weight (**a**) and food intake (**b**) in ICR mice. The results are given as the mean ± standard deviation (*n* = 8). Different letters indicate statistically significant differences between means at *p* < 0.05. CON: no exercise group, E: high-intensity exercise group, E + CRG: high-intensity exercise group supplemented with CRG, E + HRG: high-intensity exercise group supplemented with HRG.

Body weight and feed intake of mice were determined every Monday morning during the experiment periods containing adaptation period for treadmill. Body weight and feed intake of mice were not measured during the acclimation period of seven days. As shown in Figure 1a, not all of the groups did showed changes in their body weight. The amount of food intake at three weeks was decreased in all groups (Figure 1b). It is likely that this decrease can be attributed to the fact that the last week of the experimental period had a short period from Monday to Friday (sacrificed).

### 3.2. GOT, GPT, MDA, and Glycogen Analysis in Blood and Tissues

**Figure 2:**
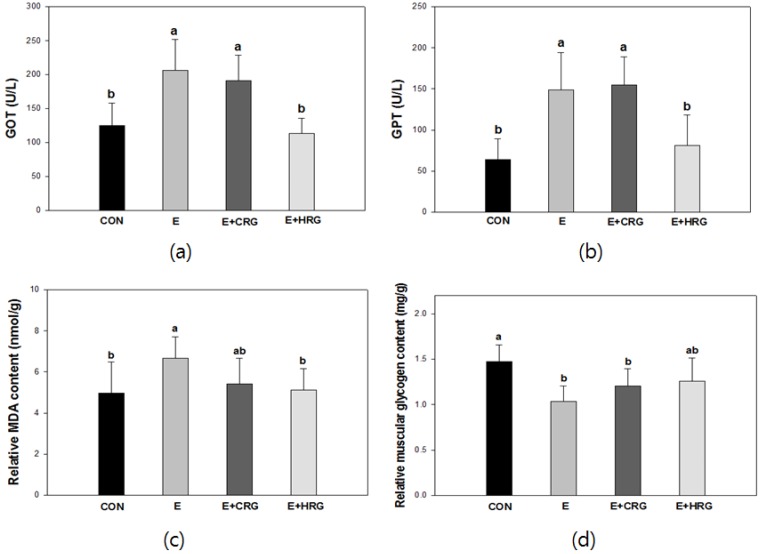
Effects of HRG on GOT (**a**), GPT (**b**), and MDA (**c**) levels and glycogen content (**d**) in blood and tissues. The results are given as the mean ± standard deviation (*n* = 8). Different letters indicate statistically significant differences between means at *p* < 0.05. CON: no exercise group, E: high-intensity exercise group, E + CRG: high-intensity exercise group supplemented with CRG, E + HRG: high-intensity exercise group supplemented with HRG.

The indices of oxidative stress in blood and tissues were analyzed (Figure 2). Figure 2a indicates that the GOT level (125 ± 32 U/L) of the CON group is statistically similar to that of HRG group (113 ± 22 U/L), which was supplemented with HRG extracts. However, the levels of GOT in both E (206 ± 45 U/L) and CRG (191 ± 37 U/L) groups were higher than those of other groups. The GPT levels of both CON (64 ± 25 U/L) and HRG (81 ± 37 U/L) were found to be significantly lower than those of E (149 ± 45 U/L) and CRG (155 ± 34 U/L) (Figure 2b). There were similar patterns between the levels of GOT and GPT in each group.

To determine the effect of HRG extracts on oxidative damage of liver, hepatic MDA levels were measured. It was observed that the MDA content of the CON group (4.9 ± 1.5 nmol/g) was the lowest, but the MDA content of the E group (6.6 ± 1.0 nmol/g) was the highest. The MDA content (5.1 ± 1.0 nmol/g) of the HRG group was similar to the CON group but the CRG group (5.4 ± 1.2 nmol/g) was higher than the HRG group (Figure 2c).

The effect of HRG extracts on skeletal muscle glycogen content was described in Figure 2d. Whereas the CON group had the highest content (1.4 ± 0.1 mg/g), other groups, such as E, CRG and HRG groups, had decreased muscle glycogen contents. However, the glycogen content (1.2 ± 0.2 mg/g) of the HRG group was statistically higher when compared to those of both CRG (1.2 ± 0.1) and E (1.0 ± 0.1) groups. There was no significant difference between E and CRG groups.

### 3.3. G6PDH and LDH Enzyme Activities in Gastrocnemius Tissue

**Figure 3:**
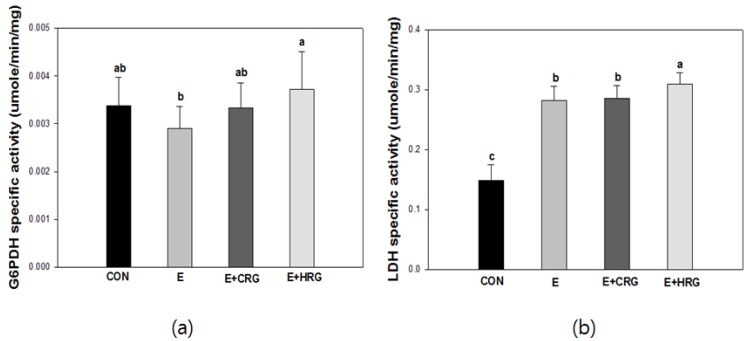
Changes of G6PDH (**a**) and LDH (**b**) enzyme activities in gastrocnemius tissue. The results are given as the mean ± standard deviation (*n* = 8). Different letters indicate statistically significant differences between means at *p* < 0.05. CON: no exercise group, E: high-intensity exercise group, E + CRG: high-intensity exercise group supplemented with CRG, E + HRG: high-intensity exercise group supplemented with HRG.

To investigate whether HRG extract has an effect on enzyme activities induced by exercise, the enzyme activities of G6PDH and LDH were determined in gastrocnemius tissue (Figure 3). The HRG group showed the highest G6PDH activity compared with E and CRG groups. Therefore, these data indicated that HRG increased the levels of G6PDH during exercise suggesting HRG may have protective effects against oxidative stress-induced muscle damage. In the case of LDH activity, HRG supplementation did not reduce the activity of LDH.

### 3.4. Cu/Zn-SOD and MRF4 mRNA Expression

**Figure 4:**
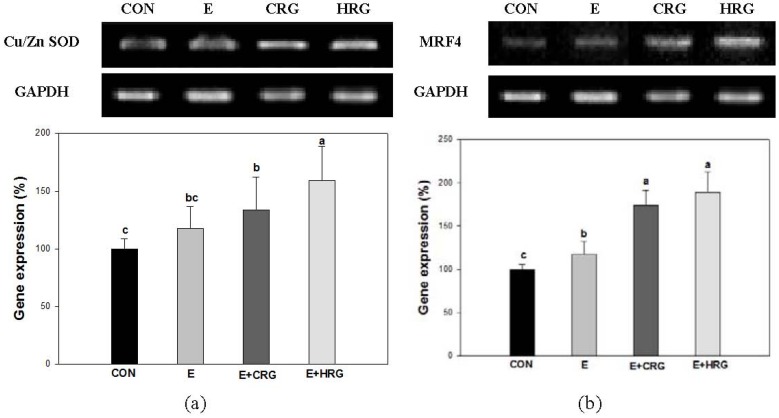
Effects of HRG on Cu/Zn SOD and MRF4 mRNA expression levels in muscle tissue. (**a**) Cu/Zn-SOD, (**b**) MRF4. The results are given as the mean ± standard (*n* = 8). Different letters indicate statistically significant differences between means at *p* < 0.05. CON: no exercise group, E: high-intensity exercise group, E + CRG: high-intensity exercise group supplemented with CRG, E + HRG: high-intensity exercise group supplemented with HRG.

In Figure 3b, HRG supplementation did not attenuate the LDH activity. Therefore, we investigated whether HRG supplementation modulates oxidative stress induced by LDH in gastrocnemius tissue. The mRNA levels of Cu/Zn-SOD and muscle regulatory factor 4 (MRF4) were measured (Figure 4). The mRNA expression levels of Cu/Zn-SOD and MRF4 in all exercise groups were increased compared to the CON group. Interestingly, the mRNA expression levels of Cu/Zn-SOD and MRF4 in the HRG group were significantly higher than those found in the CON and E groups. The mRNA expression levels of Cu/Zn-SOD and MRF4 in CRG group were higher than those of the E group.

## 4. Discussion

KGR is produced through drying and steaming processes, and is used by many Asians to improve health and physical fitness [21,22]. Recently, several studies have reported that KRG has various biological activities, such as antioxidative, anti-obesity and anti-infection activities [23,24,25]. KRG contains bioactive compounds such as ginsenosides and phenolic compounds which can vary considerably according to the conditions of the heat process [13]. For these reasons, the identification of bioactive components in KRG has been regarded as an object of scientific study. Therefore, our study investigated the antioxidative effects of HRG rich in maltol and phenolic compounds on exercise-induced oxidative stress in an experimental mouse model.

It was reported that the enzymes’ activity of GOT and GPT were increased by acute physical exercise [26]. The levels of GOT and GPT in serum of mice as well as the levels of MDA in liver homogenates are increased by oxidative stress which causes oxidative damage in liver tissue. MDA, an end product of lipid peroxidation, is used as a marker of tissue damage [27,28]. In the present study, we evaluated the indices of oxidative stress and investigated the protective effect of red ginseng against oxidative stress in serum and tissues. As shown in Figure 2, we observed significant increase in serum GOT and GPT activities in the E group. In addition, high intensity exercise led to the destruction of live tissue as the increased MDA levels in liver of the E group when compared with CON. The elevations of these markers proved that ROS induced oxidative damages in liver. HRG treatment remarkably decreased the levels of GOP and GPT in serum, whereas CRG treatment did not decrease the levels of GOP and GPT. Moreover, the hepatic MDA level of the HRG group was considerably attenuated compared with those of the E group (Figure 2c). Therefore, these results indicate that HRG has protective effect against exercise-induced oxidative stress.

We have investigated the muscular glycogen content, which plays an important role as a fuel source in muscle contraction [29]. During exercise, muscular glycogen is gradually reduced, and subsequently depleted. As a result, exercise capacity is significantly exacerbated and fatigue occurs during the exercise [30]. Figure 2d shows the muscular glycogen content of each group. The glycogen content of the E group was remarkably decreased after high intensity exercise. Interestingly, the HRG group had higher glycogen content than those of E and CRG groups. These results indicate that HRG would be more beneficial during subsequent exercise than CRG and also helpful for resistance to fatigue.

LDH induced by high intensity exercise is associated with both the generation of lactate and muscle damage [31]. Moreover, G6PDH is one of the regulatory enzymes in the pentose phosphate pathway which plays an important role in the form of nicotinamide-adenine dinucleotide phosphate (NADPH) which is needed for the homeostasis of intracellular glutathione [32]. As shown in Figure 3, the G6PDH activity of the HRG group was elevated compared to those of the E group. G6PDH is associated with the maintenance of glutathione. Therefore, these data suggest that HRG may contribute to the protective effect against oxidative stress by increasing the activity of G6PDH. In this study, the LDH activity of HRG is more increased than in other groups. It has been known that LDH generates lactate from pyruvate made from glucose via glycolysis and the generated lactate can be associated with tissue damage [33]. Therefore, we investigated both the mRNA expression levels of an antioxidant enzyme and a myogenic regulatory factor. High-intensity exercise can enhance the production of free radicals, reduce levels of antioxidant-associated enzymes in tissues and blood, and lead to tissue injury [34,35]. SOD, a family of enzymes, defends against oxygen free radicals by catalyzing the elimination of the superoxide radical [36]. Previous studies have demonstrated that administration of ginseng elevated the levels and activity of antioxidant enzyme SOD [37,38]. In line with this, the mRNA expression of Cu/Zn-SOD in the HRG group was remarkably higher than in other groups. These results indicated that the elevation in SOD expression as scavenger enzymes after red ginseng administration resulted in the decrease of MDA levels and consequently, prolonged exercise duration until exhaustion [39]. Interestingly, the mRNA expression of MRF4 in HRG group was greatly increased. MRF4 belongs to the basic helix-loop-helix class of transcription factors which influence skeletal muscle development [40]. These results support scientific claims that red ginseng has ergogenic properties in facilitating recovery from high intensity exercise [39,41]. Taken together, our results suggest that HRG has protective effects against oxidative stress induced by lactate in muscle and contributes to enhanced exercise performance ability by upregulating the mRNA expression of both Cu/Zn SOD and MRF4.

## 5. Conclusions

In conclusion, HRG protected tissue damage resulting from oxidative stress induced by high intensity exercise. Consequently, our results indicate that HRG, which contains phenolic compounds increased by high heat and high-pressure processes, may be responsible for the enhanced antioxidative activity.
